# Impacts of Clinical Decision Support Systems on the Relationship, Communication, and Shared Decision-Making Between Health Care Professionals and Patients: Multistakeholder Interview Study

**DOI:** 10.2196/55717

**Published:** 2024-08-23

**Authors:** Florian Funer, Diana Schneider, Nils B Heyen, Heike Aichinger, Andrea Diana Klausen, Sara Tinnemeyer, Wenke Liedtke, Sabine Salloch, Tanja Bratan

**Affiliations:** 1 Institute for Ethics and History of Medicine Eberhard Karls University Tuebingen Tuebingen Germany; 2 Institute for Ethics, History and Philosophy of Medicine Hannover Medical School Hannover Germany; 3 Competence Center Emerging Technologies Fraunhofer Institute for Systems and Innovation Research ISI Karlsruhe Germany; 4 Institute for Medical Informatics University Medical Center – RWTH Aachen Aachen Germany; 5 Department of Social Work Protestant University of Applied Sciences Rhineland-Westphalia-Lippe Bochum Germany; 6 Ethics and its Didactics, Faculty of Theology University of Greifswald Greifswald Germany

**Keywords:** clinical decision support system, CDSS, health care professionals, patients, relationships, communication, shared decision-making, Germany

## Abstract

**Background:**

Clinical decision support systems (CDSSs) are increasingly being introduced into various domains of health care. Little is known so far about the impact of such systems on the health care professional–patient relationship, and there is a lack of agreement about whether and how patients should be informed about the use of CDSSs.

**Objective:**

This study aims to explore, in an empirically informed manner, the potential implications for the health care professional–patient relationship and to underline the importance of this relationship when using CDSSs for both patients and future professionals.

**Methods:**

Using a methodological triangulation, 15 medical students and 12 trainee nurses were interviewed in semistructured interviews and 18 patients were involved in focus groups between April 2021 and April 2022. All participants came from Germany. Three examples of CDSSs covering different areas of health care (ie, surgery, nephrology, and intensive home care) were used as stimuli in the study to identify similarities and differences regarding the use of CDSSs in different fields of application. The interview and focus group transcripts were analyzed using a structured qualitative content analysis.

**Results:**

From the interviews and focus groups analyzed, three topics were identified that interdependently address the interactions between patients and health care professionals: (1) CDSSs and their impact on the roles of and requirements for health care professionals, (2) CDSSs and their impact on the relationship between health care professionals and patients (including communication requirements for shared decision-making), and (3) stakeholders’ expectations for patient education and information about CDSSs and their use.

**Conclusions:**

The results indicate that using CDSSs could restructure established power and decision-making relationships between (future) health care professionals and patients. In addition, respondents expected that the use of CDSSs would involve more communication, so they anticipated an increased time commitment. The results shed new light on the existing discourse by demonstrating that the anticipated impact of CDSSs on the health care professional–patient relationship appears to stem less from the function of a CDSS and more from its integration in the relationship. Therefore, the anticipated effects on the relationship between health care professionals and patients could be specifically addressed in patient information about the use of CDSSs.

## Introduction

### Background

A multitude of expectations and hopes are associated with the development of technologies to support difficult decision-making situations in health care. Clinical decision support systems (CDSSs) are seen as a possible solution for managing an exploding amount of health care data and scientific evidence in the decision-making for individual patients. In particular, the combination of advanced data storage and processing capabilities enabled by artificial intelligence (AI), or rather, machine learning, have led to a rapid exploration of the application areas in almost all health domains (eg, diagnostics, prognostics, therapeutics, and research).

The use of CDSSs is associated with ethical and social issues, such as patient safety, algorithmic transparency, lack of appropriate regulation, liability, and accountability and requires the development of a sophisticated governance approach with regard to AI-empowered health care [1-4]. The use of CDSSs also adds another layer to the health care professional–patient relationship. Therefore, it will invariably be connected with certain effects, for example, on the physician–patient relationship, that are likely to involve changes in the mode of interaction [4-6]. On the one hand, the use of CDSSs is expected to reduce workload and facilitate a more empathetic relationship with patients due to the time gained [7]; by contrast, it is feared that CDSSs will undermine the relationship of trust between patients and health care professionals, for example, through the so-called computer paternalism [4,6].

There are comparatively a large number of studies that address the potential impact of CDSSs on professionals’ clinical decision-making, in part by drawing on social epistemology or literature on professionals’ moral responsibilities [8-15]. Due to the expected impact of CDSSs on the doctor–patient relationship, some scholars suggest that it could evolve into a “doctor–computer–patient relationship” [16-18]. Illustrating this new kind of a triadic relationship, Braun et al [13] outlined three different interaction modes that illustrate how AI-based CDSSs could influence the roles of clinical agents:

1. A conventional AI-based CDSS, which supports health care professionals by generating options from patient data in the electronic health record (EHR) for professionals to consider.
2. An integrative AI-based CDSS could instead support shared decision-making between clinicians and patients, as such systems not only collect the data sets from the EHR and other patient data (thus expanding the EHR) but also present the data analysis in a way that can be used for patient interaction. The results of the algorithmic analysis not only can constructively complement the shared decision-making process but can also exacerbate existing tensions between the agents due to the lack of transparency of the AI-based CDSSs [13].
3. A fully automated AI-based CDSS that interacts directly with patients and enables them to make decisions independently and without human clinical expertise, for instance, with regard to taking medication. So far, it is unclear whether this interaction mode can contribute to increased patient participation or create new vulnerabilities, (eg, due to a lack of data literacy and biased training data) [13].

**Figure 1:**
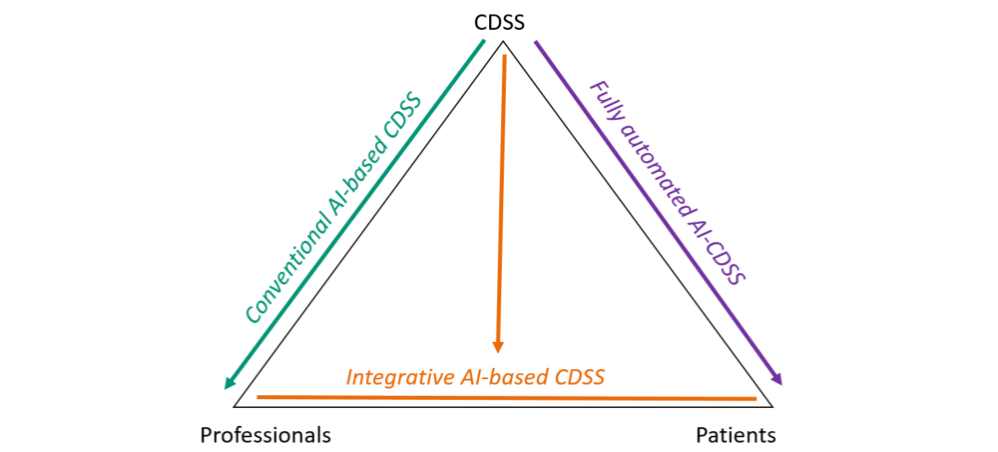
Modes of interaction illustrating how artificial intelligence (AI)–based clinical decision support systems (CDSSs) could influence the roles of clinical agents (author’s representation of the 3 types of interaction developed by Braun et al [13]).

These modes of interaction are shown in Figure 1.

### Objective

Despite this intense debate, there are only a few studies in which the empirically gathered perspectives of patients and clinicians [11,15,19-21] on a CDSS are presented together; there are exceptions such as those studies in which digital tools are intended to support the interaction between clinicians and patients, such as patient decision aids [22]. Despite the increased use of CDSSs in nursing [23-26], empirical studies with a focus on nurse–patient interactions involving CDSSs are still scarce. Therefore, the main motive for our research project was to evaluate and highlight the ethical and social aspects in terms of their importance for the various stakeholders in clinical decision-making. As part of a qualitative study combining semistructured interviews and focus groups, we explored how different stakeholders perceive the inclusion of CDSSs in decision-making processes within health care, what effects they expect for the health care professional–patient relationship, and to what extent the use of CDSSs should be addressed in information and education provided to patients. This study helps to rectify the lack of evidence in at least 3 ways: first, we explore expectations from a multi-professional perspective, interviewing both future doctors and future nurses; second, we consider the perspectives of future health care professionals and of patients; third, we include different forms of decision support for different areas of health care (ie, surgery, nephrology, and outpatient intensive care). This enables us to identify similarities and differences in the use of CDSSs in various application areas.

## Methods

### Overview

The study is part of the research consortium Decision Support in Routine and Emergency Health Care: Ethical and Social Implications (DESIREE). The aim of the research project is to explore key ethical, social, professional, and technical aspects of digital decision support in health care by combining empirical and theoretical approaches. The reporting was guided by the COREQ (Consolidated Criteria for Reporting Qualitative Research) checklist [27].

### Recruitment and Sample

The data collection was carried out between April 2021 and April 2022 as a methodological triangulation at 2 different institutions in Germany. On the one hand, individual semistructured interviews were conducted at a maximum care hospital with 15 medical students and 12 trainee nurses, all of whom were near the end of their training. In this way, individual attitudes in dealing with CDSSs could be examined in depth, and comparisons could be made between the different professions. We included people at the end of their respective training and, therefore, future health care professionals as interviewees for 2 main reasons. First, this was a relatively homogenous sample of people with the same level of medical or nursing experience, education, and familiarity with technologies in everyday life. Second, they had comparable knowledge of current technologies in the health care sector, even if only introductory, due to the content of their training. On the other hand, 3 focus groups with 18 patients (5-7 per group) were conducted by Fraunhofer Institute for Systems and Innovation Research ISI. This aimed to open up a communicative space for patients in which they could exchange opinions and viewpoints and thus foster mutual reflection as “experts in their own right.”

A convenience sampling approach was used, and respondents were included if they belonged to one of the stakeholder groups: patients who are members of self-help groups or institutions, medical students (fourth and fifth year of study), and trainee nurses (second and third year of training); were aged ≥18 years; had sufficient knowledge of German; were capable of giving consent; and were, according to their own declaration, in sufficiently good health. Since the discourse within a focus group can be considered as an expression of collective knowledge and homogeneous groups (ie, individuals with similar experiences and similar diseases) are better suited for this purpose [28], study participation was promoted in selected patient self-help groups. With the exception of 1 participant (acute hospitalization), all participants (44/45, 98%) recruited for the study took part in the interview or focus group.

There was no relationship established between participants and researchers before the interviews or focus groups. In some cases, focus group respondents knew each other. Respondents were informed in advance that the interview or focus group topic was “clinical decision support systems and digitalization in health care” but not the specific questions that were to be discussed.

### Data Collection

Due to the COVID-19 pandemic and mobility issues of some patients, all individual interviews and focus groups were conducted via video calls instead of face to face. Respondents were generally at home during the interview and focus group; most respondents were also alone unless they needed caregiver support. In such cases, the caregivers did not actively participate in the focus group. The interviewers used interview guides for the different stakeholders (refer to Multimedia Appendices 1 [15,29] and 2). As part of each interview and focus group, we used a stimulus including illustrations that introduced 3 different CDSS application scenarios. The CDSSs used as stimuli were (1) the AI-supported CKDNApp to assist nephrologists in prognosis assessment and therapy planning for patients with chronic kidney disease; (2) an AI-supported CDSS to assist surgeons with intraoperative navigation by providing recommendations for incision lines; and (3) the Safety Box, a rule-based alarm system designed to provide nurses and relatives with recommendations for action in the care of ventilated patients in the home environment. One of these CDSS examples was presented in each focus group, based on which a CDSS was used to treat their specific condition; medical students were presented with stimuli 1 and 2, and trainee nurses were presented with stimulus 3. The similarly structured stimuli allowed both a comparable understanding of the CDSS presented and, especially in the case of patients, abstraction from their own personal situation. By being confronted with the CDSS stimuli, respondents were asked about their hopes and concerns of implementing such technology, especially regarding the health care professional–patient relationship. They were asked whether and how the relationship might change and what information they thought was important to know before such a system could be deployed. From the responses, different perceptions and expectations of CDSSs can be derived, which in turn may have different implications for the health care professional–patient relationship. This also results in specific information and education needs for professionals.

Audio recordings were made to document the interviews and focus groups, and recordings were transcribed ad verbatim and pseudonymized. Field notes were taken during and after the interviews or focus groups. Data collection was terminated when theoretical saturation had been reached, that is, when additional interviews or focus groups did not provide additional information with respect to the research question.

### Data Analysis

The individual interviews lasted on average 51:26 (range 29:44-75:37) minutes; the focus groups lasted on average 114:17 (range 110:07-123:04) minutes, including a short break after 1 hour. The sociodemographic characteristics of the respondents are presented in Table 1.

The data were analyzed using structured qualitative content analysis according to Kuckartz and Rädiker [30]. The recording transcripts were assigned categories that were drawn both deductively from literature research and inductively from the interviews or focus groups themselves. Coding rules were explicated for the main categories, and examples were identified (refer to coding tree in Multimedia Appendix 3). The categories were developed and revised during the coding procedure. Two of the authors independently coded the collected data using the software MAXQDA 2020 (VERBI GmbH); one coded the transcripts of the individual interviews, and the other coded the transcripts of the focus groups. Ambiguous passages were discussed within the research team and then solved by consensus. During the coding, the focus of the analysis was directed toward distinct topics, such as role setting and relationships or patient information and education. Suitable exemplary codes were selected in order to answer the research question; these have been translated from German into English and included in this paper.

**Table 1:** Sociodemographic data of persons participating in individual interviews and focus groups (n=45).

			Medical students	Trainee nurses		Patients
Participants, n (%)			15 (33)	12 (27)		18 (40)
Age (y), mean (range)			25.5 (23-36)	25 (20-50)		45.5 (24-76)
**Gender (self-reported)**, **n (%)**						
	Men	7 (47)			2 (17)	6 (33)
	Women	8 (53)			10 (83)	12 (67)

### Ethical Considerations

This study was approved by the Research Ethics Committee of the Hannover Medical School, Germany (registration number 9805_BO_K_2021) as part of the project DESIREE. All respondents provided informed documented consent to participate in this study. At any time during the study, respondents had the opportunity to stop participating. Respondents received a small amount of monetary compensation for taking part in the interview or focus group.

## Results

### General Information

The empirical results will be presented in three thematic parts: (1) CDSSs and their impact on the roles of and requirements for professionals, (2) CDSSs and their impact on the relationship between health care professionals and patients (including communication requirements for shared decision-making), and (3) stakeholders’ expectations for patient education and information about the CDSS and its use. In the following sections, we use the abbreviations “SI” for (medical) student interviews, “TI” for (nursing) trainee interviews, and “PFG” for patient focus group.

### CDSSs and Their Impact on the Roles of and Requirements for Professionals

The respondents agreed that CDSSs should only be used to *support their decision-making*. They believed that this kind of support is provided by making expert or aggregated knowledge available to CDSS users. Some patients associated CDSSs with being able to pool expertise from different institutions (eg, across clinics), federal states (in Germany), or countries (PFG nephrology). As such, CDSSs could also be used in the training of future specialists as learning support (PFG surgery). Consequently, a CDSS is not seen as an “agent” in its own right, but only as an aid:

[I]t is also important for work that it’s still made clear: “Okay, this is not a new agent—so to speak—an independent one, but a supplementary aid to what I am doing as a doctor at this moment.”

SI5

In general, respondents understood support for decision-making as assistance of an individual professional within their decision-making rather than supporting a team. Patients emphasized that the use of CDSSs should not lead to the replacement of professionals who, for example, have more experience and can consider different factors and perspectives in their decision-making:

I think that the experience that a doctor has, I believe that nothing can replace it, not with any technological things, because everyone knows that from route guidance systems (laughing).... So I think the [the doctor’s] combination of experience is very important and that you can really see it [the CDSS] as support.

PFG surgery

Medical students, however, expected a potential replacement in fields such as radiology and claimed, for instance, that they take such considerations into account when choosing their residency training [eg, SI9].

Especially for the Safety Box to support care in home environments, patients saw the risk that the use of CDSSs could lead to a devaluation of the nursing profession if financial resources are invested more in the acquisition of technical systems such as CDSSs rather than in the training of professionals:

[It is better that the nurse] is well trained and can communicate with me when I am not able to speak or that I can interpret the alarms from the ventilator correctly, that she knows what to do, than that I bring in such a [CDSS] that thinks for the nurse, but why should it think for the nurse when the nurse has a mind of her own?

PFG home care

One concern of the professionals is a potential information overload, as they do not have the time or competences to check all the recommendations of a CDSS comprehensively, even if the CDSS refers their base of scientific evidence, research literature, or guidelines. However, if doctors can no longer keep track of large amounts of data, then they are not in a position to check for sources of error. In this sense, 1 medical student maintained the following:

[T]hen I can’t actually check this algorithm anymore or track it, to see if there’s an error in it somewhere or if data was collected incorrectly somewhere.

SI8

Some students therefore feared that they may become dependent on the algorithmic support, eventually even “helplessly in the hands of the developers of the CDSS” [SI8]. Some patients were equally concerned that the use of CDSSs would provide professionals with a false sense of security and that individual characteristics of a case might be neglected because of the CDSS recommendation obtained on the basis of probability.

Therefore, respondents emphasized that the use of CDSSs places new demands on the profession. Some students spoke of a change in professional self-image, so that responsibilities and demands on the medical profession might change and call for much more in-depth education, for example, on how data are generated and how CDSSs work in general:

[T]hat you understand what data are, how they are processed and how a kind of neural artificial network works. That you can also weigh things up and that this weighting can also be further differentiated by machine learning and that it then produces an output, which I can use afterwards for my clinical decision-making. That would also, I think, be enough for me [to know] for now.

SI8

Correspondingly, patients emphasized that professionals need to understand, comprehend, and be able to communicate information that comes from the CDSSs. They insisted that the training of professionals should include basic medical, statistical, and information technological knowledge. That should enable professionals both to evaluate CDSS information in a well-founded manner and to make clinical judgments without such support. Indeed, patients would only accept the use of CDSSs in everyday practice if well-trained medical experts were present all the time, that is, inexperienced professionals should not interact with the CDSSs on their own. Moreover, some patients expected AI-based CDSSs to be used only after professionals have had intensive training (eg, after acquiring a device operator’s license after a minimum number of training sessions).

One of the unique features of the focus groups with patients was the concern that the CDSSs could be used for other purposes in addition to improving the quality of care. On the one hand, patients feared that the use of CDSSs would be prioritized due to economic interests and that professionals could be influenced in their decision-making to deviate from their conviction about the most suitable option, for example, because only certain treatments are financially compensated (eg, PFG home care). On the other hand, patients recognized the potential for the use of CDSSs to lead to greater control of patients and their adherence to treatment, for example, by other stakeholders such as health insurers:

I was just wondering whether something like this could also arouse desires, i.e. if this data is available in such a simple form. Whether health insurance companies, for example, would come up with the idea to say: “We will only fund this medication for patients where the doctors can prove that the patients adhere well to the instructions.” In other words, it [the AI-CDSS] could be detrimental to you because there is a desire for it elsewhere.

PFG nephrology

Patients expected that such third-party interests could reduce the range of medical services actually available to them. Other interests thus become intertwined in the decision-making and interaction between patients and professionals.

### CDSSs and Their Impact on the Relationship Between Professionals and Patients

Most respondents expected changes in the relationship and communication between health care professionals and patients through the use of CDSSs. Communication was cited by the respondents as an important factor for successful treatment:

I see this as a double-edged sword. Because as we have already said a few times today, that this communication with the affected person, yes, that is the key. And not just in the situation where the person needs me, but maybe even also during the rest of the time.

PFG home care

The respondents distinguished 3 possible scenarios in their representations. First, some respondents (particularly trainee nurses) were concerned that the use of CDSSs would lead to an increasing focus on technology because professionals would “cling very much to the technology” [SI10] or “just concentrate on the screen” [TI3], leading to reduced attention to patients. Patients had similar concerns, fearing a maldistribution of attention:

I see it similarly to [R7] that I always say to nurses: “Look into my eyes first and see how I make gestures and perceive, and don’t just worry about the beep or the device.” Exactly, so I find this [people-to-people] contact much, much more important.

PFG home care

Some patients feared that the use of CDSSs could undermine trust and interaction; this is not limited to the cases where a CDSS has a control function (eg, nonadherence of therapy measures by patients). They feared that health care professionals’ actions could be reduced mostly to basic care:

We do not want to be called or seen as a “living object,” so to speak. We want professionals to communicate with us, maybe in a different way than at the moment, but we don’t want them to just.... I mean, I don’t even want to compare it to an animal. But that they just feed us and then it’s enough. Then you can move away, because the technology is at work, and then I don’t have the connection to caregivers anymore.

PFG home care

Since they linked CDSSs with the tendency toward the standardization of treatment, patients on a ventilator at home feared that patient autonomy could be impeded by the use of CDSSs. They associated standardization in nursing above all with making services more economical, which would then be less flexible in their response to individual patient needs. Therefore, they were concerned about an increase in computer paternalism:

There is always a very big risk of being impinged upon by such a device.

PFG home care

In addition, particularly home care patients emphasized that the “right to be medically unreasonable” must still exist even with the implementation of CDSSs. They associated this with the right to be able to make independent decisions that go beyond defined medical limits, in case of doubt, even against both medical necessity and the recommendation of the CDSS:

We also have a right to medical irrationality, and this is of course completely undermined by such a device. If the device says: You’re over-breathing, you’re getting too much air... and the person themselves says: “Yes, I know, but I need this because I can speak better that way.” Then there is already a conflict: Am I allowed to act against the medical advice of this device? But that is also a conflict for the nurse. Okay, do I follow what the patient says or what the device says?

PFG home care

Second, some respondents recognized the potential for CDSSs’ visualizations to contribute in a beneficial way, for example in the case of the CKDNApp for communicating prognostic statements or in the case of the Safety Box for explaining professional actions and their necessity to patients. Although the benefit of using visuals for shared decision-making is not unique to the use of CDSSs, such ideas were enough for many medical students to assume that the use of CDSSs would lead to a necessity for increased communication in the future: on the one hand, the “increased communication” brought about by the use of CDSSs can strengthen shared decision-making, since the respondents expected that communication about CDSS results would be regularly included in future discussions between patients and professionals. On the other hand, it would also lead to additional effort, since the necessity to communicate the results and their interpretation could come to the fore, even if there is limited time available. In particular, applications that potentially involve patients would require professionals to communicate more frequently with their patients about how certain CDSS results are obtained, how they are to be interpreted, and what implications they may have on the patient’s circumstances.

The inclusion of CDSSs in the shared decision-making process is seen as problematic in certain situations, particularly if professionals are unable to assess the appropriateness of a specific CDSS recommendation for a patient (refer to the CDSSs and Their Impact on the Roles of and Requirements for Professionals section). If the clinical judgment of the professional does not match with the recommendation of the CDSS, some medical students see the only reasonable solution in communicating both the CDSS recommendation and their own recommendation to the patient, thereby invoking the principle of “patient autonomy” [SI6]:

Then I would say both, as I always think that patients should be informed, and they should be informed throughout, all the way across the spectrum, which means you should give them all their options and I think patients should be able, I mean, responsible adult patients, should be able to make their own decisions and figure out for themselves where they put their trust. So, I would say: “I did this with an app [CKDNApp] with a certain data set that stated, your prognosis is rather poor. I personally using my clinical experience would say I don’t see it as pessimistically, but the bottom line is it’s hard to say.” So, I would list both options and then it is up to the patient...and then the patient can think for himself, whom or what he trusts more

SI4

This way of dealing with such challenges was not an isolated case. Indeed, most patients also expected professionals to be transparent about whether and how CDSS recommendations had been incorporated into their professional decision:

Yes, I think it’s better if the doctor tells me, because then I know whether it comes from him/her, from his/her experience, from his/her many years of experience with patients, or simply from this app, from mathematical things, etc., from different values.

PFG nephrology

According to the respondents, the involvement of CDSSs in decision-making processes could also lead to a loss of trust in the health care professional–patient relationship, if it is somewhat unclear to patients where a recommendation for therapy comes from:

Yes, I find it a bit difficult when the doctor says that everything is fine now, for example, I think she/he has to tell me that it comes from her/him or from this app. So I don’t know if the trust is still 100% there when this app is involved.

PFG nephrology

However, some medical students also felt that communicating divergent recommendations to patients would be inappropriate. They feared additional confusion on the part of the patients because they would have difficulty deciding which recommendation they should follow.

While 1 medical student surmised that patients would generally trust the recommendation of the human more than that of the CDSSs, this was far from clear to patients. They argue that trusting someone is also a conscious decision, regardless of whether you trust people or technical systems. Some patients perceived the recommendations from CDSSs as a second opinion. If the professional judgment and the recommendation of the CDSS should diverge more frequently, some patients feared that they might get into a conflict of loyalty:

And if the doctor would say to me: “The app recommends that... but I would like to tell you something completely different.”; and I would always follow the app, then I would actually get into a predicament somehow, it would be about me and the doctor as individuals. If I were to keep saying: “I don’t trust you like I trust the app, so please do it the way the app says,” then I think I would get into difficulties with the doctor at some point.

PFG nephrology

Finally, 1 patient changed their opinion during the focus group, as they felt that this complete transparency had not been provided so far either:

Well, for me it wouldn’t be important, I’m realizing in the course of the evening. The doctor also seeks advice from studies, for example, and then doesn’t tell me every time: “There is a new study on which I am now basing my opinion, where there are new findings.”

PFG nephrology

Third, some respondents associated the use of CDSSs with the opportunity for patients to be more actively involved in decision-making and therapy planning. For example, in the case of the CKDNApp, patients assumed that their own role in the treatment process could be strengthened by contributing independently to the collection of data:

I think it would be good if you, as a patient, could see at least partial data [of the CDSS] yourself, in order to be involved, in order to be taken seriously as the main point of focus.

PFG nephrology

Some patients, therefore, interpreted the inclusion of CDSSs in the decision-making process as a form of patient empowerment. In addition, some respondents emphasized that the patient’s wishes and values should be the focus of shared decision-making processes; they, therefore, demanded that CDSSs should take patient wishes into account in the algorithmic data processing. This is especially the case for legally binding statements or the living wills of patients:

Well, I just think it’s really important that when such [CDSS] systems are set up, that it’s really not just about the trivial data and facts, but everything really has to be looked at carefully: Is there a living will and what are the wishes? And that this should not be ignored under any circumstances. I find this very important in this context.

PFG home care

As such, CDSSs could be understood as an advocate for patients in the context of treatment. Some patients associated CDSSs as an instrument to counter the omnipotence of professionals in the event of possible treatment errors; for example, if therapeutic decisions were automatically recorded by the CDSSs, this would allow the decisions and actions of professionals to be revealed retrospectively (PFG home care and PFG surgery).

### Expectations for Patient Education and Information About CDSSs

Regarding the need for the information about CDSS use, a broad consensus can be found among medical students and patients that, at the current state, patients should definitely be informed and that informed consent is required for CDSS use. Only trainee nurses were reluctant to take a stand on this issue as they viewed patient information and education as primarily the doctors’ responsibility.

Despite the widespread agreement that patients should generally receive information about CDSS use from professionals, there was disagreement among professionals about the extent of information required. Some medical students emphasized that the requirements for patient education and information depended on the individual needs and wishes of patients:

Even if you explain therapies or diagnostic tools, the patients’ necessity for information is very different. Some want to know in great detail what happens when and how and what that means and what effects it has, and others somehow fall back into this paternalistic pattern: “I’m in a bad way, you tell me what I should do, I’ll do it and then I’ll feel better.”

SI11

This perception is consistent with the statements made by the patients of the focus groups, who, on the one hand, wanted to be informed about the use of CDSSs, but on the other hand, also emphasized that this does not apply to all patients equally:

It’s probably going to vary from patient to patient. But there are also people who are not interested [in informed consent] at all, right? They say right here: “Give me the piece of paper, I’ll sign it, I couldn’t care less.” So, I don’t think you’ll be able to reach a consensus; one wants to know and the other honestly doesn’t give a damn.

PFG surgery

The respondents argued that the use of CDSSs presents clinical practice with the challenge of figuring out which information patients do and do not want. Some patients even articulated their right not to know:

[T]hat possibly the patient and the doctor have a dialogue about what is displayed [from the CDSS] to the patient and what is not. So, for example, if I don’t want to know what my vascular risk is, that the doctor then switches it off for me. That would be personalized, I would think, that would be very nice if that was done, because it’s just a thing that patients want.

PFG nephrology

Other medical students doubted the feasibility of being able to give patients detailed information. According to some, patients do not need to be informed about how an AI-based CDSS works and how the data are generated. Giving detailed rationales is not common in other clinical decision contexts (eg, in performing an operation), and most patients would not be interested in such explanations anyway.

Indeed, for some medical students, the need to provide patients with information decreases as the system is increasingly used to support a decision that the health care professionals would normally make on their own. For example, 1 medical student saw only a limited necessity to provide patients with detailed information using conventional CDSSs, as long as “it is a *support* system and you [as doctor] do not hand over the competence” (SI5).

In principle, respondents agreed that a minimum of information about the benefits, risks, and limitations of CDSSs “as with other treatments” would be necessary. Particular attention should be paid to the presentation of “failure rates” and “success rates” of CDSSs, that is, false-negative and false-positive outcomes (compare with eg, SI13*;* also PFG nephrology), which should also be contrasted with alternative well-established procedures for better interpretation. Of particular importance to medical students and patients was the need to communicate CDSS recommendations as probabilities that can be contextualized, especially in the case of prognosis and treatment planning based on those recommendations:

Everything is only a probability and that would have to be clearly communicated. So, you would have to explain exactly to the patient with this app [= CKDNApp]: “Well, what the app shows here is only a probability or the most probable progression, but this probability is not a fixed item, it is a range.... And of course, that is always developing, but what really happens in the future, I can’t promise anything.” I can only draw conclusions with the knowledge from the past and make assumptions for the future and I would present them to her like that.

SI8

Some medical students believed that providing knowledge about the underlying probabilities is necessary to give patients an idea of how the decision support impacts their own actions:

[B]ut I would try to give him [the patient] a basic understanding of what exactly the [CDSS] shows me and to what extent the [CDSS] has the power to make decisions, or I would make it clear to him that at the end of the day I still control the cutting tools or whatever, and that the [CDSS] doesn’t do that.

SI10

To this end, communication skills should be trained in order to be able to “adequately capture and also explain” (SI5) the functions of CDSSs and “to interpret and explain them [the outcomes] to the patient” (SI8). Similarly, 1 patient wanted their doctors to “be fit in communicating scientific data to nonscientifically literate people” (PFG nephrology) when using the CKDNApp.

In the light of previous experience with inadequately “informed consent,” 1 patient emphasized the desire for comprehensible support materials that explain CDSSs:

One thing that would be important to me, however, is that there should be a comprehensible declaration of consent, so, in plain language. I think that these consent forms are often undervalued by the medical side, so, you often get a piece of paper that you sign, and it says: “You had no more questions.” And you couldn’t really ask any questions at any point. I’ve also experienced something like that I’ve already had to sign it before I’ve even seen a doctor.

PFG nephrology

Some medical students and trainee nurses emphasized that providing such background information is not only a necessary prerequisite for making informed decisions but also has a reassuring effect on patients and can help to reduce fears and reservations regarding CDSSs or digital technology in general.

## Discussion

### Interpretation of Findings and Comparison With the Literature

Although the study participants were presented with 3 examples of a “conventional AI-based CDSS” [13] (Figure 1), in which primarily professionals interact with the CDSS, it was noticeable that the respondents expanded the scenarios and CDSSs presented at different points to include their own set of associations. For example, in the case of the CKDNApp, patients expected that they would interact directly with the system by providing the CDSS with further records; and in the case of the Safety Box, they expected it to bring greater control to the care relationship and the care providers’ actions. These broader associations could be crucial because the respondents mainly addressed the challenges and issues that Braun et al [13] associated primarily with integrative AI-based CDSSs, such as problems of transparency, power, and loyalty conflicts in the health care professional–patient relationship. The authors stated that “integrative AI-based CDSSs” run the risk of exacerbating existing tensions between those involved since it is unclear to what degree or in which order the existing records of both the actors are integrated into the analyses and recommendations of the AI-based CDSSs. Although the study participants highlighted the supportive nature of CDSSs in decision-making processes, the respondents, first and foremost the patients, anticipated that CDSSs would disrupt the usual health care professional–patient relationship, representing some kind of a third party. This perception as a “third party” who “creates the image of another person in the room” [11] is already known from previous studies and applications [31-34].

Our findings suggest that the involvement of CDSSs could be associated with a restructuring of established power and working relationships between health care professionals and patients in at least three ways.

First, some respondents feared that technology could dictate to its users what has to be done (for “computer paternalism” and restrictions of autonomy, also refer to Čartolovni et al [4], Lorenzini et al [18], and Rajpurkar et al [35]). This would be accompanied by significant changes in their own position in the decision-making process. Patients feared that the weight given to their statements in the decision-making process would be less and that their preferences would more frequently be ignored or given less consideration [36,37]. Health care professionals, in turn, feared that they would have less autonomy in decision-making about the most appropriate method or procedure from a medical point of view and thus become more vulnerable for litigation, especially in those situations where they do not adhere to the CDSS recommendations.

Second, some respondents could imagine the involvement of CDSSs in the shared decision-making process (eg, as a form of presentation [38]). In this context, respondents often presupposed that the recommendations of the CDSSs should be made transparent to the patients by the professionals [11]; however, the degree to which it was necessary for professionals to present information and how transparent this information should be were also critically questioned. Worries about patients being overloaded or overinformed played a role here. Patients viewed the involvement of CDSSs as a “second opinion” in addition to the opinion of professionals, which would not always be beneficial, leading to possible conflicts of loyalty.

Third, some respondents expected that the use of CDSSs could also empower patients and allow them to participate more actively. This included, for example, the notion of CDSSs acting as an advocate for patients by taking patients’ wishes into account when recommending therapies and preventing them from being bypassed or even documenting or reporting potential treatment errors by professionals.

The evaluation of the possible effects and changes to the health care professional–patient relationship that could arise with the use of CDSSs are ambivalent; this is not only because they represent extreme positions (ie, empowerment of patients vs computer paternalism) but also because of the associated uncertainty with regard to the extent to which the integration of the CDSSs impacts the interaction between health care professionals and patients in the shared decision-making process. For our respondents, and especially for the patients, it was unclear what role the CDSSs should play: while on the one hand, CDSSs are described by some respondents as merely supportive tools within the decision-making process, on the other hand, some believe that the CDSSs can challenge the professionals’ expertise, for example, by being perceived as a “second opinion” alongside that of the professional (refer elsewhere for more empirical data and the conceptual discussion about “second opinions” [12,14,15,18,39-43]). The fact that this view is also held by the professional trainees should be a cause for concern. They were themselves undecided about how to evaluate the recommendations of the CDSSs and how to deal with divergent recommendations in the case of conflict and even leaving it up to the patients themselves to decide how the CDSS recommendations should be integrated, if necessary (in this sense, refer also to Heyen and Salloch [6], Grote and Nucci [9], and Funer [12]). Given that patients are generally less knowledgeable about CDSS recommendations, how they are generated, and what their clinical-therapeutic significance is, as reflected in their desire for simple and understandable CDSS recommendations, patients should not be given the responsibility of evaluating the CDSS recommendations by themselves.

An interesting aspect is the difference in the perception and assessment between the different groups of professionals, that is, prospective clinicians and nurses. Whereas the former, in their comment on the changed relationship, focused on the increased need for communication and information that would accompany the use of CDSSs [19], the latter were primarily concerned about changes in personal care and attention economy [37], which could be to the disadvantage of patients.

Current studies show that professionals are concerned that digitalized processes could increase rather than decrease the daily workload [4,19,44,45]. Our study confirms these concerns insofar as the respondents expected time-consuming communication with regard to the CDSS, its functioning and its recommendations, for which they do not yet feel adequately prepared (a self-perceived lack of “IT maturity” is also reported by Frisinger and Papachristou [19]). Nevertheless, a similar need was already predicted in the German Technology Radar 2021: “professionals will increasingly have the task of clearing up misunderstandings in the case of supposed or incorrect information, interpreting and communicating the results of automated diagnoses, relativizing or explaining acquired knowledge” [44] (author’s translation). Accordingly, the respondents in all stakeholder groups deemed it necessary that the professionals should be sufficiently prepared as part of their training and studies.

The adequate training of professionals stems specifically from the need to be able to ultimately provide robust support to patients, enabling them to make decisions that reflect their own treatment preferences. According to the respondents, this requires a strong focus on communication, as this represents a central aspect of the professional–patient relationship [18,19]. In the interviews, this communication mostly centered around providing patients with information about the use of CDSSs. There was a consensus among all stakeholder groups that CDSSs should only be used after informed consent has been obtained. The exact amount of information must be adapted to the individual patient and situation. Whether it is sufficient to merely discuss the advantages and disadvantages of CDSS use (eg, in the form of comparable error and success rates, which can be contextualized and compared with alternative treatment options), or whether the way in which CDSS function and, where applicable, the quality of their underlying data need to be the subject of the information is a matter of considerable controversy among all the respondents, both patients and professionals. Whatever the case, this requires sufficient knowledge on the part of professionals in dealing with the systems and their respective limitations [10,18]. In addition, it requires specific communication skills in order to be able to explain to patients the results [11,18], such as, the value of statistical algorithmic statements and how to interpret them, in an appropriate way. In terms of organization structure, time is needed for the extra effort of communication [19]. Information materials could also play a practical role in improving communication.

It may be surprising that the expected changes in the health care professional–patient relationship and their implications for potentially difficult decision-making situations have hardly any impact on the content of patient information about CDSSs to date; however, this might be explained by the fact that these aspects have, to date, not been an element of patient information and education and are therefore not associated with this format. The study results made it clear that embedding the CDSSs can take place or be designed in different ways and thus cannot be derived from the function of the CDSSs alone. However, our study results also highlighted that an exchange of ideas about the future professional–patient relationship is important in order to address undesirable implications and fears about CDSS use at an early stage and, if necessary, to counteract them. According to some interviews, this should result in a kind of “meta-communication,” by jointly working out which information the patient does and does not want and which decisions should be made to what extent by whom. While this can hardly be provided within the usual time and framework of patient information by the professionals alone, the professionals represent an important contact for the patients to address such aspects in the future.

### Limitations of the Study

Due to the study design, there were some interpretative limitations of the results, the most important of which will be briefly mentioned here. Convenience sampling and participant acquisition by self-help groups probably led to the inclusion of particularly committed and interested patients. It can be assumed that the patients of the focus groups were particularly likely to be actively included in their own therapy; the opinions of patients who are less interested in shared decision-making might thus be underrepresented. Taking this into account, no peer review and member check according to Lincoln and Guba [46] could be done for this study. Moreover, the information that the study would be about digital forms of decision support was provided before the study; this might have especially motivated the participation of those who were already quite open-minded about technology use or have strong opinions on it. The statements obtained from the individual interviews and focus groups thus represent central aspects of the stakeholders interviewed, which can also be generalized to a certain extent for these groups; however, the statements do not meet the requirements for being representative.

Further limitations result from the fact that the “professionals” interviewed were students and trainees at the late stage of their qualification. Although they are mostly familiar with the status quo of the latest developments in medicine and nursing and already have practical experience, their statements do not reflect the attitudes of advanced practitioners with extensive clinical practice; in this regard, reference should be made to other existing empirical studies whose results complement our findings [11,19-21,37].

There were also inherent limitations in the selection of the case studies. While these served as stimuli to concretize possible fields of application of CDSSs and, thanks to their uniform structure, enable a comparison across the various stakeholders, they also focused the respondents’ perception on certain decision situations and thus potentially influenced them during the interview or focus group (eg, through a strong focus on the case study). Furthermore, the fact that in some cases, respondents discussed their ideas on the topic using a familiar decision support system (eg, a satnav device) as a comparison for the CDSS presented or made assumptions about other features may have contributed to respondents misunderstanding the specific nature of the CDSS presented.

Finally, the statements compiled here reflect the associations and attitudes of the respondents reflecting on how they would perceive and evaluate the potential changes in the relationship, communication, and shared decision-making. Consequently, the respondents were speaking about hypothetical situations and not directly from their own experience. However, associations and attitudes do not allow us to draw conclusions about how they will actually occur in the future. Instead, they provide indications of what is important to the interviewees, from a current perspective, for their future work in health care. In turn, this allows appropriate measures to be taken to avoid or counteract many of the concerns right from the outset [47].

### Conclusions

The roles of health care professionals and patients, and therefore also their relationships, are in a state of flux, evolving within the wider context of trends such as economization or quality assurance requirements. Our empirical research suggests that the use of CDSSs is likely to restructure established power and working relationships between health care professionals and patients. In this respect, the interviewed stakeholders not only fear the possible introduction of computer paternalism and expect multiple influences and substantial changes in the shared decision-making processes but also hope for the promotion of active participation in the sense of patient empowerment. To prevent the expected negative effects and enable positive effects, the interviewees emphasized that the use of CDSSs would require increased communication, for example, to understand the systems used, the scope and extent of their use, and the impact on specific decision-making situations; this would involve more time spent on patient education and ongoing communication. Our results shed new light on the existing debates by showing that the expected impact of CDSSs on the relationship appears to stem less from the function of a CDSS and more from the way of their integration in the health care professional–patient relationship. Ultimately, our results suggest that whether CDSSs will have an overall positive or negative impact on the health care professional–patient relationship will depend on the extent to which such impacts are considered in the design and even more the use of CDSSs. This includes, for example, raising awareness through patient information and professional training.

Our study makes a significant contribution to the discussion of CDSSs in routine and emergency care, since it empirically examines the potential implications on the relationship level and is consistent with comparable studies. The findings underline the importance of the relationship level in the use of new technology, such as CDSS, both for patients and professionals.

## Appendix

Multimedia Appendix 1 Interview guides for interviews with health care professionals.

Multimedia Appendix 2 Interview guides for interviews with patients.

Multimedia Appendix 3 Coding tree and rules.

## Abbreviations

AI: artificial intelligence
CDSS: clinical decision support system
COREQ: Consolidated Criteria for Reporting Qualitative Research
DESIREE: Decision Support in Routine and Emergency Health Care: Ethical and Social Implications
EHR: electronic health record
PFG: patient focus group
SI: student interview
TI: nursing trainee interview

## Data Availability

The data are not publicly available as they might compromise research participants’ privacy and consent.
