# Supplementary material for: Impacts of Clinical Decision Support Systems on the Relationship, Communication, and Shared Decision-Making Between Health Care Professionals and Patients: Multistakeholder Interview Study
Source: J Med Internet Res. 2024 Aug 23;26:e55717. doi: 10.2196/55717 (PMC11380058; doi:10.2196/55717)
Supplement: Multimedia Appendix 1 [file jmir_v26i1e55717_app1.pdf]

This supplement was previously published in: Funer F, Liedtke W, Tinnemeyer S, et al. Responsibility and decision-making authority in using clinical decision support systems: an empirical-ethical exploration of German prospective professionals' preferences and concerns. *Journal of Medical Ethics*. Published Online First: 22 May 2023. doi: 10.1136/jme-2022-108814

Supplement 1: Medical students' interview guide and presentation of the CDSS used

**Guiding question(s)**	**Aspects to be addressed/ concrete demands**	**Maintenance issues/ control issues**
**Topic complex I: Entry**		
Dear Sir/Madam... Nice of you to take the time to talk to me today about digital decision support systems in healthcare. Thank you very much for your time.<br><br>If you like, we can be on a first-name basis. Is that okay with you?<br><br>Your participation in this interview is, of course, voluntary and you can withdraw your consent to participate in the study at any time without giving any reason. We will record the interview so that I can concentrate on it better, and an external transcription office will then transcribe it. The interview data will then be analyzed by our study team – of course, with strict confidentiality and nondisclosure. When we publish our results, there are only a few, short quotes (usually single sentences) in the publications. However, readers will never be able to attribute the quotes to a person (i.e. data are anonymous to readers). (Attribution is possible within the research team using a pseudonymization list – as long as it exists.)<br><br>Are you okay with me recording our conversation from now on?<br>*[If participant agrees, turn on the recorder – if not, take notes.]*<br><br>So, our conversation is now being recorded. We have already talked about the fact that your participation in this research project is, of course, voluntary. You can stop or interrupt the interview at any time. Feel free to go into as much detail as you see fit in the conversation; we have time. There are no wrong or right answers. We are interested in *your* experiences and assessments, which means that you are now the expert. You can be absolutely honest; we will not judge any answers.<br><br>Do you have any questions about the interview process?<br><br>Initial question: To what extent is the topic "digitization in medicine" an issue for you at all (studies, exchange with fellow students, etc.)?		

This supplement was previously published in: Funer F, Liedtke W, Tinnemeyer S, et al. Responsibility and decision-making authority in using clinical decision support systems: an empirical-ethical exploration of German prospective professionals' preferences and concerns. *Journal of Medical Ethics*. Published Online First: 22 May 2023. doi: 10.1136/jme-2022-108814

Supplement 1: Medical students' interview guide and presentation of the CDSS used

**Topic complex II: Operating room of the future/surgery navigation**		
[*Presentation of case vignette 1.*]		*Can be used for all topic complexes:*
Please describe your first thoughts about this system.	What do you spontaneously find good about using that system during surgery compared to surgery without the support of that system?<br><br>And what do you spontaneously find less good or even bad about using this system?	Can you describe this in more detail?<br><br>Why don't you tell me a little bit more about it?<br><br>Can you give an example of…?
To what extent would you like to use this system yourself in your daily work in the future?	What would be necessary for you to use this system yourself? (e.g. training, fitting into workflow, ...)	Would/Did that matter to you?<br><br>What happened next?
Imagine that the system gives you an incision recommendation which you follow, but you hit a nerve tract, causing incontinence in the patient. Please describe your thoughts on this scenario!	Imagine that the patient now asks you what exactly happened and how it could have happened. What do you tell your patient?<br><br>To what extent should your patient understand the system?<br><br>To what extent should you inform your patients that they will be operated on using this system? Does your patient have to consent to the use of the system?<br><br>To what extent would you feel responsible for the consequences of the surgery?	What do/did you think?<br><br>Can you think of anything else to say about that?<br><br>How did it actually come about that ...?

This supplement was previously published in: Funer F, Liedtke W, Tinnemeyer S, et al. Responsibility and decision-making authority in using clinical decision support systems: an empirical-ethical exploration of German prospective professionals' preferences and concerns. *Journal of Medical Ethics*. Published Online First: 22 May 2023. doi: 10.1136/jme-2022-108814

Supplement 1: Medical students' interview guide and presentation of the CDSS used

Imagine that the system recommends you a cutting line that you think is too risky. How do you behave in this situation?	To what extent would you hold others responsible? Do other people/instances (e.g. developer, system, hospital) play a role?<br><br>Under what conditions would you not follow the system's recommendation? What does it take for you to trust the system?	
**Topic Complex III: Nephrologists prognosis and therapy planning app**		
[*Presentation of case vignette 2.*]<br><br>Please describe your first thoughts about this app.<br><br>To what extent would you like to use this system yourself in your daily work in the future?<br><br>Imagine that the app predicts a very favorable progression of the disease for your patient. In reality, however, the disease progresses extremely poorly and your patient has to go on dialysis regularly just a few weeks after your conversation. What are your initial thoughts on this scenario?	What do you spontaneously find good about using this app?<br><br>And what do you not find so good or even bad?<br><br>Are there any requirements?<br><br>The patient asks you how exactly this incorrect prediction of the progression of the disease could have come about. How do you answer your patient?<br><br>To what extent should your patient understand the system?<br><br>To what extent should you inform your patients that the app is affecting your recommendations? To what extent would you feel responsible for the incorrect prognosis?	

This supplement was previously published in: Funer F, Liedtke W, Tinnemeyer S, et al. Responsibility and decision-making authority in using clinical decision support systems: an empirical-ethical exploration of German prospective professionals' preferences and concerns. *Journal of Medical Ethics*. Published Online First: 22 May 2023. doi: 10.1136/jme-2022-108814

Supplement 1: Medical students' interview guide and presentation of the CDSS used

Imagine that the nephrology app predicts that your patient's disease will progress much worse than you think. Why don't you describe how you would deal with this situation?	To what extent would you hold others responsible? To what extent do other instances/people play a role?<br><br>Which progress of the disease do you report to your patient?<br><br>Should you tell your patient about both progressions of the disease? Imagine that the patient asks you which progression (s)he is more likely to get. What do you answer?<br><br>What does it take for you to trust the system?	
**Topic complex IV: Training**		
What do you think you need to be able to work well with such systems as the ones presented?<br><br>To what extent do you feel prepared to use such systems yourself?	To what extent do you think you should understand the system or the app?<br><br>How did your studies contribute to your preparation?<br><br>What would you like to see in your studies to improve your preparation?<br><br>What would you like to see in your future postgraduate residency training?<br><br>What do you think about acquiring the necessary competencies alongside your job?	

This supplement was previously published in: Funer F, Liedtke W, Tinnemeyer S, et al. Responsibility and decision-making authority in using clinical decision support systems: an empirical-ethical exploration of German prospective professionals' preferences and concerns. *Journal of Medical Ethics*. Published Online First: 22 May 2023. doi: 10.1136/jme-2022-108814

Supplement 1: Medical students' interview guide and presentation of the CDSS used

	Is "learning by doing" enough for you or do you want further training?<br><br>How has your private use of digital devices contributed to your preparation? (Do you perceive differences within your own generation?)	
**Other**		
We need data sets – e.g. patient data – for the development of clinical decision support systems. To what extent do you see your future task in contributing to good data sets?<br><br>Is there anything else you'd like to get off your chest that we haven't addressed yet?		
**Sociodemographic checklist**		

1. Gender
   - ♀
   - ♂
   - other
2. May I ask your age?
3. How long have you been studying human medicine? In which semester are you?
4. Have you already completed other training/courses of study?
   - No
   - Yes: which ones?
5. Have you gained practical experience independently of your course of study?
   - No
   - Yes: in which area?

This supplement was previously published in: Funer F, Liedtke W, Tinnemeyer S, et al. Responsibility and decision-making authority in using clinical decision support systems: an empirical-ethical exploration of German prospective professionals' preferences and concerns. *Journal of Medical Ethics*. Published Online First: 22 May 2023. doi: 10.1136/jme-2022-108814

Supplement 1: Medical students' interview guide and presentation of the CDSS used

Presentation of "Operating room of the future" (computer-based + ML-supported assistance system, surgical navigation device)

- I would like to introduce you to a decision support system whose field of application is in surgery; I would be interested in your opinion about it.
- To illustrate, I'll show you a short video [play entire video]: https://www.youtube.com/watch?v=jJDANlCdMCg&t=155s
- I repeat and add: the decision support system is intended to assist the surgeon during surgery by indicating an optimal incision line (incision direction and risk structures that must not be violated).
- Objective: avoidance of patient harm, shortening of the operating room time.
- This can be used when a rectal resection must be performed on a patient diagnosed with rectal cancer and can be performed laparoscopically (laparoscopy – reference to video).
- The decision support system helps surgeons remove pathological tissue, preserve healthy tissue and not injure nerve pathways or major vessels during surgery by displaying the incision line + risk structures.
- Thus, there are no functional restrictions, such as incontinence or impotence, as a result of the surgery in the patient (which are otherwise common in rectal surgery).
- This is especially helpful in difficult surgical phases, for example, in the case of poor visibility due to adhesions or for inexperienced surgeons.
- Suggestions for incising are displayed to surgeons on a screen (as in the video), alternatively, they can also work with digital glasses (system as a supplement to DaVinci).
- How is this assistance function created? (Function: show the best cut and certain risk structures that must not be violated).
    - AI (specifically: machine learning) is used to create the assistance function
    - Here, a system learns to perform these functions using examples from the past
    - What examples are used to learn here?
        - Surgical videos of the Da Vinci Surgical System
        - Individual images are isolated from these videos and then manually entered on them, which is what you see there (e.g. where is the large intestine, where is the small intestine, where are nerves to be spared, where is the narrow area where cutting is allowed)
        - The system is fed with these images and learns in this way "like a toddler" (e.g. what a large intestine looks like)
        - We cannot say what exactly the system has learned to distinguish the colon from the non-colon (we have only fed the system with pictures on which information is noted, such as "this is the colon")
    - The system learns in this way where risk structures are and how deep a surgeon may cut
    - During surgery, the system receives live images of the patient's abdomen, plus preoperative images, and compares these with what it has learned in advance
    - It then shows the surgeon the optimal incision line or even the location of important nerves on the screen or data glasses – i.e. the system superimposes this information on the camera data of the abdomen of the patient lying on the operating table

This supplement was previously published in: Funer F, Liedtke W, Tinnemeyer S, et al. Responsibility and decision-making authority in using clinical decision support systems: an empirical-ethical exploration of German prospective professionals' preferences and concerns. *Journal of Medical Ethics*. Published Online First: 22 May 2023. doi: 10.1136/jme-2022-108814

Supplement 1: Medical students' interview guide and presentation of the CDSS used

Presentation of the CKDNapp (Chronic Kidney Disease Nephrologist's App)

- I would like to introduce you to a clinical decision support software that is provided for smartphones, tablets and PCs, and can be used in the internet browser or as an app.
- This is another example of one of many decision support systems for doctors and I would be interested in your opinion on it.
- This decision support system is designed to assist nephrologists in the care of patients with chronic renal failure.
- It is well-known that chronic renal failure is one of the most common causes of death and a very complex disease, the progression of the disease is very individual and there are many concomitant diseases.
  - The current condition and expected disease progress of a patient with chronic renal failure depends on numerous different parameters (e.g. demographic, disease history, lifestyle and medication parameters)
  - Traditional and novel biomarkers can also provide information about future condition of the patient
- In order to provide optimal care to patients with chronic kidney disease, physicians must collectively evaluate and integrate all of these disparate and complex data based on medical knowledge (only then can therapy be personalized).
- The system supports the physician in the complex process of data integration and, thus, in the personalized treatment of patients.
- I would like to describe to you the decision support system in more detail with help of this graph (see right).
  - The patient diagnosed with renal insufficiency consults a nephrologist
  - Diagnosis by nephrologist: (S)he takes the medical history and performs further examinations (e.g. urine examination, blood test)
  - The nephrologist then enters the patient data into the software
  - In addition: all available laboratory test results, if necessary, from other specialists

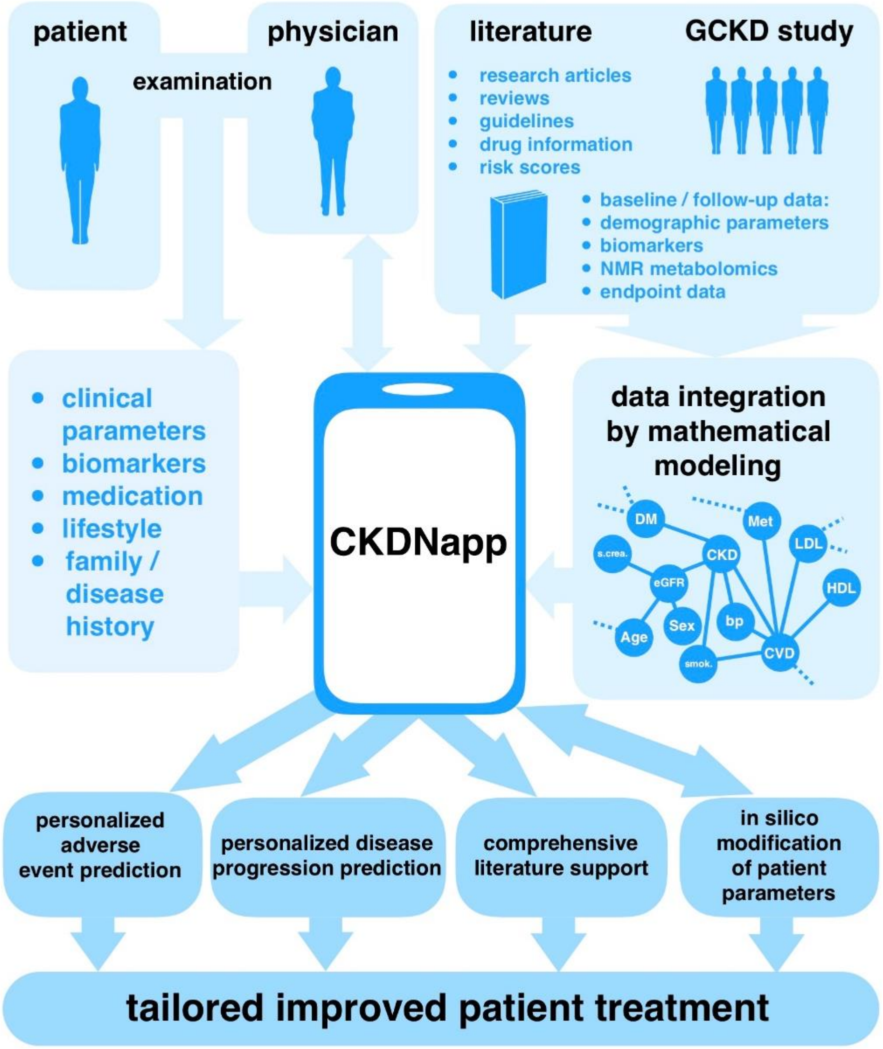

Graph 1: Schematic workflow of CKDNapp's development and application (cf. online: https://www.sys-med.de/de/juniorverbuende/ckdnapp/). Copyright: Michael Altenbuchinger and Helena U. Zacharias.

This supplement was previously published in: Funer F, Liedtke W, Tinnemeyer S, et al. Responsibility and decision-making authority in using clinical decision support systems: an empirical-ethical exploration of German prospective professionals' preferences and concerns. *Journal of Medical Ethics*. Published Online First: 22 May 2023. doi: 10.1136/jme-2022-108814

Supplement 1: Medical students' interview guide and presentation of the CDSS used

- CKDNapp uses these data to match them with results from digital databases (parameters of other patients) and digital libraries; here, complex algorithms work in the background. The App is built on two pillars (see diagram):
  - First pillar: comprehensive mathematical diagnostic and prediction models (e.g. for personalized prediction of cardiovascular events, end-stage renal failure or patient death)
    - These models are learned using data from the "German Chronic Kidney Disease" study, for example, demographic, clinical data, biomarker data or metabolome data (i.e. the elaborate study of biochemical metabolites)
    - Integration of all these patient parameters is performed using the latest machine learning methods
    - (Focus on metabolism: metabolites are intermediate/final products of the body's metabolism and are found in all body fluids/tissues. The kidney is one of the main regulators of metabolism. If kidney function is disturbed, metabolism becomes disordered and is then reflected in an altered composition of numerous metabolites in the blood/urine. Metabolites can serve not only as biomarkers for the patient's current physiological state, but also to predict future events)
    - The German Chronic Kidney Disease is one of the world's largest observational studies of kidney disease with over 5,000 patients followed for over 10 years
    - The mathematical models learned are fed into the app in a fixed way (not the data from study)
  - Second pillar: Comprehensive collection from already identified risk predictors found in the scientific literature
  - Based on these two pillars, software can enable various functions:
    - It provides personalized predictions for adverse medical events and disease progression (tells us probabilities for e.g. acute renal failure, cardiovascular or cerebrovascular events, death and complications, such as gout flares) (currently we are talking about a 4-year period); CKDNapp also shows which predictors are behind this evaluation
    - Extensive literature support (research articles, guidelines, drug information, established risk scores)
    - The software enables *in silico* changes of patient parameters (virtually change patient parameters and simulate the resulting disease development: this means digital testing of lifestyle adaptations, such as weight loss or smoking cessation).
- I would like to demonstrate one more core element of the decision support system: a calculator to estimate the individual risk of developing end-stage renal disease requiring dialysis or kidney transplantation within one, two, three or four years.
  (The interviewer opens the risk calculator [https://ckdn.app/tools/eskdcalc/], enters values [first using the default settings, then changing values – creatinine value first to 6 milligrams per deciliter, then to 13] and presents the results.

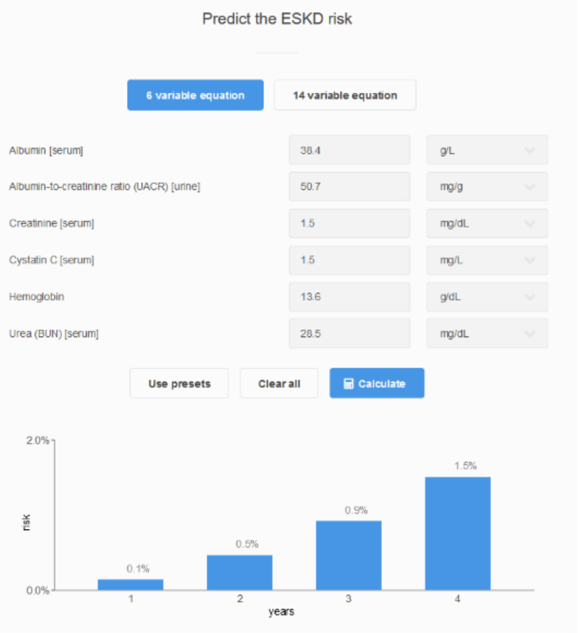

Graph 2: Exemplary CKDNapp's result (cf. Zacharias, H.U., et al.: A predictive model for progression of CKD to kidney failure based on routine laboratory tests. *American Journal of Kidney Diseases* 79,2 (2022): 217-230). https://ckdn.app/tools/eskdcalc/

This supplement was previously published in: Funer F, Liedtke W, Tinnemeyer S, et al. Responsibility and decision-making authority in using clinical decision support systems: an empirical-ethical exploration of German prospective professionals' preferences and concerns. *Journal of Medical Ethics*. Published Online First: 22 May 2023. doi: 10.1136/jme-2022-108814

Supplement 1: Prospective nurses' interview guide and presentation of the CDSS used

**Guiding question(s)**	**Aspects to be addressed/ concrete demands**	**Maintenance issues/ control issues**
**Topic complex I: Entry**		
Dear Sir/Madam... Nice of you to take the time to talk to me today about digitalization and digital decision support systems in healthcare. Thank you very much for your support of our research.<br><br>If you like, we can be on a first-name basis. Is that okay with you?<br><br>Your participation in this interview is of course voluntary and you can withdraw your consent to participate in the study at any time without giving any reason. We will record the interview so that I can concentrate on it better, and an external transcription office will then transcribe it. The interview data will then be analyzed by our study team – of course, with strict confidentiality and nondisclosure. When we publish our results, there are only a few, short quotes (usually single sentences) in the publications. However, readers will never be able to attribute the quotes to a person (i.e. data are anonymous to readers). (Attribution is possible within the research team using a pseudonymization list – as long as it exists.)<br><br>Are you okay with me recording our conversation from now on?<br>*[If participant agrees, turn on the recorder – if not, take notes.]*<br><br>So, our conversation is now being recorded. We have already talked about the fact that your participation in this research project is, of course, voluntary. You can stop or interrupt the interview at any time. Feel free to go into as much detail as you see fit in the conversation; we have time. There are no wrong or right answers. We are interested in *your* experiences and assessments, which means that you are now the expert. You can be absolutely honest; we will not judge any answers.<br><br>Do you have any questions about the interview process?		

This supplement was previously published in: Funer F, Liedtke W, Tinnemeyer S, et al. Responsibility and decision-making authority in using clinical decision support systems: an empirical-ethical exploration of German prospective professionals' preferences and concerns. *Journal of Medical Ethics*. Published Online First: 22 May 2023. doi: 10.1136/jme-2022-108814

Supplement 1: Prospective nurses' interview guide and presentation of the CDSS used

**Topic complex II: Digitalization in healthcare and nursing**		
Let's start in general and you simply report what comes to your mind: to what extent is the field of "digitalization in care" a topic for you at all?<br><br>(*Focus on the use in patient care if the interview partner does not come up with this on their own*.)<br><br>What opportunities do you think digitalization will bring to care?<br><br>(*If no examples are given by the interviewee, give three different examples: nursing robot, bed exit system, hospital information system*.)<br><br>What risks and fears do you think digitalization will bring to care?<br><br>How will your profession change by the use of modern technologies?	How did you encounter the topic in the context of your training? In theory? In practice? Exchange with classmates/colleagues? Free time?<br><br>Do the opportunities or the risks predominate for you? Are you more positive or negative minded about digitalization in care?<br><br>What tasks that used to be the job of nurses can you think of that would be better handled with the help of modern technology?	*Can be used for all topic complexes:*<br><br>Can you describe this in more detail?<br><br>Why don't you tell me a little bit more about it?<br><br>Can you give an example of…?<br><br>Would/Did that matter to you?<br><br>What happened next?<br><br>What do/did you think?<br><br>Can you think of anything else to say about that?<br><br>How did it actually come about that ...?
**Topic Complex III: DSS for home respiratory care (the "Safety Box")**		
[*Presentation of the Safety Box (long-term use, emergency situation).*]<br><br>Please describe your first thoughts about this system.<br><br>To what extent would you like to use this system yourself in your daily work in the future?	What do you spontaneously find good about using this app? [*Hopes*]<br><br>And what do you not find good or even bad about using this system? [*Concerns*]<br><br>Are there any requirements?	

This supplement was previously published in: Funer F, Liedtke W, Tinnemeyer S, et al. Responsibility and decision-making authority in using clinical decision support systems: an empirical-ethical exploration of German prospective professionals' preferences and concerns. *Journal of Medical Ethics*. Published Online First: 22 May 2023. doi: 10.1136/jme-2022-108814

Supplement 1: Prospective nurses' interview guide and presentation of the CDSS used

Imagine you are caring for three patients in a respiratory care residential group. Now that the safety box has been introduced, you are no longer responsible for three patients, but five. How comfortable would you feel with this situation?	All five safety boxes show no alarm signals. How reassured do you think you would be if you left the residential group?	
	To what extent should you inform your patients, if they are conscious, about the use of the safety box?	To what extent do patients need to understand the system?
	To what extent must the patient or the legal representative give his/her consent?	
	If something goes wrong, i.e. the safety box does not trigger an alarm even though the situation actually requires it, who do you think should bear the responsibility for the consequences?	To what extent do others bear responsibility/parts of responsibility? Does the developer or the system itself play a role?
Imagine that the safety box repeatedly sounds an alarm and prompts you to check the connection of the ventilation hose. However, you do not identify a disconnection of the ventilation hose or any other hazardous situation. What should you do?	In the end, do you rely more on your assessment or on the safety box?	
To what extent do you think the use of such a system will have an impact on the relationship between you as a nurse and the patient?		
To what extent would it make a difference to you whether this system is used for the care of patients in respiratory care residential groups or for the care of patients in their own home environment?	Where would you rather use the system?	
What impact do you think will the use of such a system in nursing have on the labor market?		
**Topic complex IV: Training**		

This supplement was previously published in: Funer F, Liedtke W, Tinnemeyer S, et al. Responsibility and decision-making authority in using clinical decision support systems: an empirical-ethical exploration of German prospective professionals' preferences and concerns. *Journal of Medical Ethics*. Published Online First: 22 May 2023. doi: 10.1136/jme-2022-108814

Supplement 1: Prospective nurses' interview guide and presentation of the CDSS used

What do you think a nurse needs to be able to do in order to work well with systems like the one presented?<br><br>To what extent do you feel prepared to use such systems yourself?	To what extent do you think a nurse should understand the system?<br><br>How did your training help to prepare you?<br><br>What would you like to see in your training in terms of better preparation?<br><br>What do you think about acquiring the necessary skills while working? Is "learning by doing" enough for you or do you want further training?<br><br>How has your private use of digital devices contributed to your preparation?	
**Other**		
Is there anything else you'd like to get off your chest that we haven't addressed yet?		
**Sociodemographic checklist**		
1. Gender<br>○ ♀<br>○ ♂<br>○ other<br>2. May I ask your age?<br>3. How long have you been in nursing training?<br>4. Have you already completed other training/courses of study?<br>○ No<br>○ Yes: which ones?<br>5. Have you gained practical experience independently of your training?<br>○ No<br>○ Yes: in which area?		

This supplement was previously published in: Funer F, Liedtke W, Tinnemeyer S, et al. Responsibility and decision-making authority in using clinical decision support systems: an empirical-ethical exploration of German prospective professionals' preferences and concerns. *Journal of Medical Ethics*. Published Online First: 22 May 2023. doi: 10.1136/jme-2022-108814

Supplement 1: Prospective nurses' interview guide and presentation of the CDSS used

Presentation of the "Safety box" (not self-learning, purely rule-based)

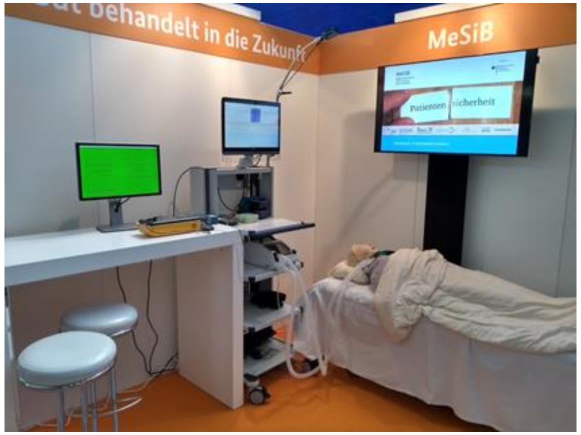

Photograph 1: Photograph of the "Safety Box" setting.

- I would like to introduce you to a decision support system that can be used in home respiratory care.
- Objective: care of ventilated patients – usually high-risk patients – even more safely in their home environment and to relieve nursing staff (or family caregivers).
- It's used for tracheotomy patients, i.e. patients who are ventilated via an artificially created tracheal opening (with no to little self-breathing).
- The system recognizes medical emergencies in the home environment (e.g. disconnection of the ventilation tube), reacts to this with an alarm and instructions for action for the nursing specialist (or for the caring relatives); in certain cases the home emergency call dispatcher is informed.
- The probability that patients suffer serious harm (e.g. hypoxia) decreases.
- With the help of this photo, I would like to give you a better understanding of the decision support system: [*Interviewer shows Photograph 1*.]
    - The photo was taken at a congress, normally the system has its place in the ventilated patient's home or room.
    - We see a bed on which the patient is lying.
    - Ventilation tubes lead away from and towards the patient, connected to the ventilator on one side and to the tracheostoma (surgically created opening of the trachea) on the other side.
    - To the left side of the bed you can see the ventilator, which can be used to make various settings (how much oxygen the patient receives, what intensity, etc.).
    - In the top corner you see a small presence sensor (thermal imaging camera), which is mounted so that the patient and people in the immediate vicinity are detected.
    - Above the ventilator you see a monitor on which you can read various parameters (e.g. heart rate, ventilation parameters, blood pressure) – I will show you exactly what you can read in a moment.
    - The small white-green box between the monitor and the ventilator is the safety box, which receives all important metrics and information:
        - Metrics of the home ventilator: information about the settings of the ventilation parameters, the parameters currently present, information about the oxygen saturation, ventilation frequency
        - Information from the motion sensor: number of people present in the room and time information (how many people were in the room how many minutes ago)
        - Information about which activities were last performed on the patient (nursing actions are not entered in the safety box by the nurse, safety box is not a documentation system; images from thermal imaging camera provide information about actions of those present – images are stored for a defined period of time)
        - Blood pressure values via blood pressure monitor

This supplement was previously published in: Funer F, Liedtke W, Tinnemeyer S, et al. Responsibility and decision-making authority in using clinical decision support systems: an empirical-ethical exploration of German prospective professionals' preferences and concerns. *Journal of Medical Ethics*. Published Online First: 22 May 2023. doi: 10.1136/jme-2022-108814

Supplement 1: Prospective nurses' interview guide and presentation of the CDSS used

- The safety box evaluates all these data and gives a recommendation on possible courses of action based on algorithms (sequence of unambiguous instructions that enables computer to solve problem). [*Interviewer shows Photograph 2*.]
  - In an emergency situation a normal alarm tone of the safety box is heard, in addition, the nurse receives instructions for action
  - Such an instruction can be, for example, the request: "Please check the connection to the ventilation tube"; such emergency measures can be read on the monitor, but we also get verbal instructions via an audio track; these instructions give emergency measures (which should stabilize the condition of the patient, maintain the circulation, etc.)
  - Thus, there is no long search for causes of problems; the nurse is immediately shown what to do in this emergency situation
  - In addition, you can see the following on the monitor in this emergency: [*Interviewer shows Photograph 3*.]
  - We can read that there is a life-threatening emergency
  - Ventilation is not ensured because the tube system is leaking, oxygen saturation drops below the alarm limit (red lung), there is no person in the room (last one left the room 10 minutes ago), no information about the respiratory rate, battery charge of the ventilator 180 minutes, heart rate and blood pressure are normal
  - "Call triggered: Safety box automatic": Here you can set in advance who should be informed by a call in a triggering situation or in an emergency – you can set a direct connection to the home emergency call, to the caregiver or to the family member.
  - Examples of other possible dangerous situations and decisions for action that can be identified or specified by the system: 1) "Please use suction because the ventilation hose is blocked," 2) "Please check the cuff pressure," 3) "Ventilation problem, please remove the machine and use a resuscitator bag," 4) "Cardiovascular failure, please perform cardiac massage and place a board under the chest" (in some cases, very precise instructions for the procedure)
- When using the "Safety Box," nurses should reflect their actions, not implement recommendations one to one.
- So far, there is only a display on the ventilator that indicates, for example, increased ventilation pressure, but gives no indication of the cause. The "Safety Box" shows the nurses which pressure increase is due to which causes
- Due to the high time requirement, respiratory care is increasingly shifting to shared apartments, where there is no one-to-one care, i.e. there are even fewer nurses who can be reached at the same time

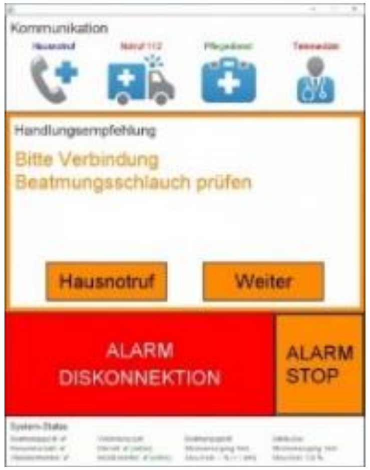

Photograph 2: Diagram with instructions in case of emergency.

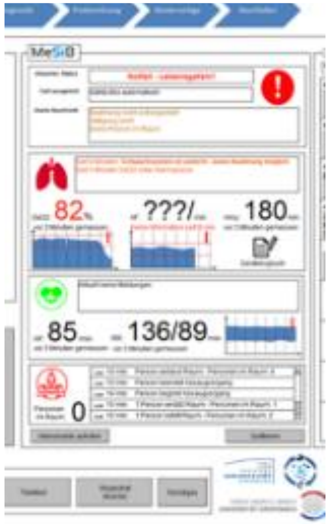

Photograph 3: Data in case of emergency.
