# Supplementary material for: Impacts of Clinical Decision Support Systems on the Relationship, Communication, and Shared Decision-Making Between Health Care Professionals and Patients: Multistakeholder Interview Study
Source: J Med Internet Res. 2024 Aug 23;26:e55717. doi: 10.2196/55717 (PMC11380058; doi:10.2196/55717)
Supplement: Multimedia Appendix 3 [file jmir_v26i1e55717_app3.docx]

**Supplement 3: Coding tree and rules**

| **Overarching categories** | **Coding rules** |
| --- | --- |
| **“Expectation of positive effects”** | The category “Expectation of positive effects” is coded when statements are made about …  … positive aspects (e.g. benefits, hopes, wishes) of digitization in general and  … positive aspects particularly of the DSS (presented). |
| **“Expectation of negative effects”** | The “Expectation of negative effects” category is coded when statements are made about …  … negative aspects (e.g. disadvantages, dangers, fears) of digitization in general and  … negative aspects particularly of the DSS (presented). |
| **“Reliability of the technology”** | The category “Reliability of the technology” is coded when statements are made about …  … the correctness of the technical analyses;  … faulty analyses and the handling of them; and  … the trust in technical systems or about aspects of a trustworthy technology (e.g. diversity/non-discrimination/fairness, robustness/security, cf. guidelines of the EU Commission). |
| **“Traceability/Comprehensibility of decisions”** | The category “Traceability/Comprehensibility” is coded when statements are made about …  … the weighting/importance of traceability in decisions in general;  … the importance of traceability in decisions made by people (physicians/nurses);  … the importance of traceability in decisions made on the basis of algorithmic analyses; and  … measures how traceability could be established (e.g. transparency, explicability of algorithmic results, explanations about medical treatments). |
| **“Trust in human actors and institutions”** | The category “Trust (in human actors and institutions)” is coded when statements are made about …  … the expectation that an event or action important to the speaker, which is in the context of digitized medicine/nursing (especially DSS use), will occur or be carried out in a way desired by the speaker but, at the same time, not controlled;  … expectations of medical and nursing staff themselves, and relevant institutions (e.g. facility providers, manufacturers, legislators); and  … someone else who has or should have such expectations (e.g. when medical students talk about their future patients). |
| **“Trust in/reliance on technical systems”** | The category “Trust in/reliance on technical systems” is coded when statements are made about …  … trust in/reliance on technical systems; and  … the necessary preconditions for trust in/reliance on technical systems (if they do not directly concern “reliability” or “comprehensibility”), e.g. individual experience with the CDSS. |
| **“Decision-making authority”** | The category “Decision-making authority” is coded when statements are made about …  … whether the recommendation of the DSS or the professional’s own judgment (especially in the case of disagreement) is given greater weight; and  … how to proceed if the professional’s assessment differs from that of the DSS. |
| **“Responsibility”** | The category “Responsibility” is coded when statements are made about …  … who (why) assumes or should assume responsibility for (the consequences of) (medical or nursing) actions influenced by a DSS. |
| **“Competencies”** | The category “Competencies” is coded when statements are made about …  … competencies required by medical or nursing staff for dealing particularly with the DSS presented;  … competencies that medical or nursing staff acquire or should acquire for digitized medicine or digitized care;  … the understanding of the technologies that medical and nursing staff and patients (should) have;  … competencies that patients need in the context of digitized medicine or digitized care; and  … competencies that patients need particularly in the context of the DSS presented. |
| **“Role setting”** | The category “Role Setting” is coded when statements are made about …  … the doctor-/nurse-patient relationship in the actual or imagined setting with and without modern technologies (incl. DSS);  … (possible) effects of the use of DSS on the doctor-/nurse-patient relationship;  … the (expectations of) roles of the human actors in the setting (doctors, nurses, patients, relatives, etc.); and  … role changes of the human actors in the setting (doctors, nurses, patients, relatives, etc.). |
| **“Patient education”** | The category “Patient education” is coded when statements are made about …  … whether and how patients should be informed in advance about the use of DSS;  … whether patients should consent to the use of DSS or if they may refuse the use of DSS; and  … what patients should be told in the event of harm in the context of the use of a DSS. |
| **“Comparison between DSS and other systems/instruments”** | The category “Comparison between DSS and other systems/instruments” is coded when statements are made about …  … to what extent the DSS presented differs from other systems and instruments used in the medical or nursing context; and  … to what extent one of the DSS presented differs from the other DSS presented. |
| **“Other”** | The category “Other” includes other aspects that are not included in the categories above, but which are related to the topic of DSS. |
